# Pulmonary vein anatomy addressed by computed tomography and relation to success of second‐generation cryoballoon ablation in paroxysmal atrial fibrillation

**DOI:** 10.1002/clc.23163

**Published:** 2019-03-13

**Authors:** Bart A. Mulder, Meelad I. H. Al‐Jazairi, Bauke K. O. Arends, Niels Bax, Leonard A. Dijkshoorn, Uzaifa Sheikh, Eng S. Tan, Ans C. P. Wiesfeld, Robert G. Tieleman, Rozemarijn Vliegenthart, Michiel Rienstra, Isabelle C. van Gelder, Yuri Blaauw

**Affiliations:** ^1^ Department of Cardiology University of Groningen, University Medical Center Groningen Groningen The Netherlands; ^2^ Department of Cardiology Martini Hospital Groningen The Netherlands; ^3^ Department of Radiology University of Groningen, University Medical Center Groningen Groningen The Netherlands

**Keywords:** anatomy, atrial fibrillation, computed tomography, cryoballoon ablation, outcome, pulmonary veins

## Abstract

**Background:**

Cryoballoon isolation is considered a safe and effective treatment for atrial fibrillation (AF). However, recurrence of AF after first cryoballoon ablation occurs in ~30% of patients. Pre‐procedurally identifying patients at risk of AF recurrence could be beneficial.

**Hypothesis:**

Our aim was to determine how pulmonary vein (PV) anatomy influences the recurrence of AF using the second‐generation cryoballoon in patients with paroxysmal AF.

**Methods:**

We included 88 consecutive patients with paroxysmal AF undergoing PVI procedure with a second‐generation 28‐mm cryoballoon. All patients were evaluated at 3, 6 and 12 months using a 12‐lead ECG and 24‐hour Holter monitoring. PV anatomy was assessed by creating three‐dimensional models using computed tomography (CT) segmentations of the left atrium.

**Results:**

Fifty‐one patients (61%) had left PVs with a shared carina, 35 patients (42%) had a shared right carina. Nine patients (11%) were classified having a right middle PV. In total 17 (20.2%) of patients had a left common PV. At 12 months, 14 patients (17%) had experienced AF recurrence. Neither PV ovality, variant anatomy, the presence of shared carina nor a common left PV was a predictor for AF recurrence.

**Conclusions:**

No specific characteristics of PV dimensions nor morphology were associated with AF recurrence after cryoballoon ablation in patients with paroxysmal AF.

## 1 INTRODUCTION

Atrial fibrillation (AF) is the most encountered arrhythmia in clinical practice. In 2010, it was estimated that 33.6 million people suffer from AF worldwide.^1^ Recent guidelines state that pulmonary vein isolation (PVI) is considered first choice treatment.^2^ PVI using cryoballoon is a common used ablation strategy, however, a significant proportion of patient experience AF recurrences.^3^, ^4^

Ablation outcome may be affected by clinical factors, such as type of AF, left atrial (LA) size, comorbidities or operator experience.^5^, ^6^ Besides these clinical characteristics, it has been suggested that PV morphology may also affect outcome. PV anatomy is very heterogeneous and optimal circumferential contact between the cryoballoon and PV/atrial myocardium is important for long‐term freedom from AF.^7^ Previous studies on the relationship between PV ovality and cryoballoon ablation showed that a larger ovality index of the left inferior pulmonary vein (LIPV) has been found to predict AF recurrence in a group of paroxysmal and persistent AF patients.^8^, ^9^ However in previous studies both the first‐ and second‐generation balloon with different balloon sizes (23‐ and 28‐mm) were used^9^ and a heterogeneous group of patients (both paroxysmal and/or persistent AF) were included.^8^

The aim of the present study was to assess PV morphology and variants including carina width, shared carina or PV ovality index and whether this is of influence on AF recurrence after PVI with the second generation 28 mm cryoballoon in a homogenous population of patients with paroxysmal AF.

## 2 METHODS

### 2.1 Patients

Eighty‐eight consecutive patients with drug‐refractory paroxysmal AF underwent second‐generation cryoballoon ablation between February 2014 and February 2015 in the University Medical Center Groningen. To ensure a homogenous group of patients, we included only patients with paroxysmal AF who underwent second‐generation cryoballoon ablation therapy in this study. Of these patients, 84 had computed tomography (CT) scans prior to the ablation procedure that could be analyzed for PV dimensions and morphology and were included in this analysis. All data were retrospectively collected from the patients' medical files. All patients consented to the ablation procedure, all data is anonymously gathered retrospectively and no additional studies were performed, therefore, no ethical approval was obtained.

### 2.2 CT image acquisition and analysis

Cardiac CT scanning was performed with a first generation dual‐source CT system (SOMATOM Definition, Siemens Healthcare, Forchheim, Germany). Scan acquisitions were made with the patient at inspiratory breath hold. A topogram was made to determine the scan range, from a couple of centimeters above the carina to just below the heart, to include all PVs entering the left atrium. CT scan protocols details include: prospective ECG triggering, sequential mode with collimation 2 × 64 × 0.6 mm using a flying z‐spot, tube voltage 100, 120, or 140 kV (depending on patient size), gantry rotation time 330 ms, reference tube current 220/200/196 mAs/rotation, respectively, electrocardiography (ECG_‐gated tube current modulation. The contrast agent injected was iomeprol 350 or 400 mg/100 mL (Iomeron 350 or 400, Bracco Imaging S.p.A., Milan, Italy). The contrast bolus depended on patient size and length of the scan, and was followed by a saline flush. Contrast‐enhanced cardiac CT scanning was performed at 70% of the cardiac cycle. Computed tomography angiography (CTA) data were reconstructed with as 1.2 mm consecutive slices in the axial plane, using a smooth kernel (B30f). The cardiac radiologist performed reporting in clinical setting, and reported on the lung vein anatomy, left atrium and thrombus in left atrial appendage on the cardiac CT, as well as side findings. For further analysis, CT scans were uploaded to Syngo X Workplace VB21C (Siemens AG, Wittelsbacherplatz, Munich, Germany) and segmented to obtain the LA. Subsequently, the scans were transferred to CartoMerge (Biosense Webster, Diamond Bar, California) for segmentation and analysis in the three‐dimensional field. The vein ostia were identified as the visual anatomical entry point of the PVs in the atrial wall, which was the point with the highest inflection.^10^ Ostium of all PVs was identified and thereafter assessment of carina width, vein diameter, and morphology. The patient population was divided into two groups and analyzed by four independent researchers, two for each group. Thus, each CT was analyzed twice by two independent researchers who were blinded to the outcome of the clinical procedure. The order of CT scans was shuffled, to account for a learning curve. The average of these measurements was calculated and used in the analysis. In case of disagreement about morphology, the case was discussed among the researchers until a consensus was reached.

The carina was defined as the segment connecting ipsilateral adjacent PVs and was measured using the shortest possible line through two points at the highest inflection of this portion (carina width). A shared carina was defined as a carina width of <5 mm. The ostium was assessed using multiplanar reformatting, measuring the largest (Dmax), and smallest (Dmin) diameter of each PV entering the LA. The ovality index was then calculated using the formula Dmax/Dmin. Veins were classified as round (ovality index <1.2), oval (1.2‐1.4) or flat (>1.4).^11^, ^12^ Left common pulmonary vein (PV) was defined as the presence of bifurcated PVs entering the left atrial contour together and a distance between the virtual border of the left atrium and the bifurcation of both PVs ≥5 mm.^13^

### 2.3 Cryoballoon ablation procedure

The ablation procedure was performed under conscious sedation. LA access was achieved with a single transseptal puncture. The target ACT level was >300. The second‐generation 28‐mm cryoballoon (Artic Front Advance, Cryocath) was used for ablation. At least two cryothermal applications (lasting 240 seconds) were delivered to isolate each vein. During cryothermal ablation of the right PVs, diaphragmatic stimulation, using a quadripolar catheter placed in the superior caval vein, was performed to avoid phrenic nerve injury. Electrical isolation of the PVs was evaluated using the circular Achieve mapping catheter (entrance block). In case of presence of remaining PV potentials pacing within the PVs was performed with 10 mA to check for exit block.

### 2.4 Post‐ablation management and follow‐up

Three outpatient clinic visits were scheduled to follow the patients at 3, 6, and 12 months after the cryoballoon procedure, each preceded by a 12‐lead ECG and 24‐hour Holter monitoring. During these visits, medical history was obtained and physical examination was performed. Patients presenting with AF symptoms were given an event recorder to document possible recurrences. In addition, information about AF, atrial flutter, or atrial tachycardia recurrence documented by the general practitioner, during emergency room visits or during hospital admissions was also collected. Antiarrhythmic medication was continued during the first 3 months after the procedure, and then discontinued in patients without symptoms of AF.

### 2.5 Covariate definitions

Time since first AF diagnosis was defined as the time from first documented AF episode till ablation date. Coronary artery disease was defined as history of myocardial infarction, percutaneous coronary intervention, or coronary artery bypass grafting. Hypertension was defined as systolic blood pressure >140 mm Hg and diastolic blood pressure >90 mm Hg or by use of antihypertensive medication. Body mass index (BMI) was calculated as the ratio of weight (in kilograms) to height (in meters) squared (kg/m^2^). Diabetes was defined as a fasting plasma glucose ≥7.0 mmol/L, a non‐fasting plasma glucose ≥11.1 mmol/L, or use of anti‐diabetic drugs. AF was considered paroxysmal if all past AF episodes ended within a week of onset (self‐terminating or by cardioversion). CHA2DS2‐VASc was defined according to the European Society of Cardiology guidelines.

### 2.6 Endpoints

The primary endpoint was the first recurrence of AF, atrial flutter, or atrial tachycardia documented by ECG, Holter monitoring (episode lasting for more than 30 seconds) or by an event recorder, or repeat ablation during a follow‐up of 12 months, excluding recurrences occurring in the first 90 days (blanking period).

### 2.7 Statistical analysis

Normally distributed continuous variables were given as mean ± SD, skewed data as median with interquartile range and categorical data as numbers with percentages. Normal distribution of data was checked using Shapiro‐Wilk W test for normal data. The χ^2^ was used to compare nominal variables. Intraclass coefficient (ICC) estimates between raters were calculated based on a mean‐rating (*k* = 2), consistency agreement, two‐way mixed effects model. The first occurrence of the primary outcome was assessed by Kaplan‐Meier curves. Univariate screening using Cox regression analysis was done to identify predictors of AF recurrence. A univariate *P* < 0.2 was required for entrance in a multivariate model. All tests of significance were two‐sided, with *P*‐values of <0.05 assumed to indicate significance. Data were analyzed with Stata version 13.0 (StataCorp, College Station, Texas).

## 3 RESULTS

### 3.1 Patient population

In total, 84 patients with paroxysmal AF were included for the present analysis. In our study population, 49 patients (58%) were male and median age at inclusion was 59 ± 10 years. AF duration was 2.9 (1.2‐6.6) years. A total of 47 patients (56%) had hypertension, 11 (13%) had diabetes mellitus, and 10 (12%) had coronary artery disease. Table 1 shows detailed patient characteristics. Total procedure time of the cryoballon ablation was 118.9 ± 37.3 minutes. The total number of cryo applications was 9.6 ± 2.7. Electrical isolation of the PVs was achieved in 90.5% of patients. In total, 6 complications occurred (data not shown): 2 (2.4%) phrenic nerve palsy (at discharge), 2 (2.4%) femoral pseudoaneurysm, 1 (1.2%) arterio‐venous fistula, and 1.2% dissection of iliac vein.

**Table 1:** Baseline characteristics

	Overall (n = 84)	No recurrence (n = 70)	Recurrence (n = 14)	*P*‐value
Age, mean ± SD	59 ± 10	58 ± 10	61 ± 9	0.32
Male sex, no. (%)	49 (58%)	41 (59%)	8 (57%)	0.92
Body mass index, mean ± SD	28 ± 5	28 ± 5	29 ± 6	0.65
Years of AF history, median	2.9 (1.2–6.6)	2.8 (1.2‐6.9)	3.2 (0.8‐5.0)	0.70
Hypertension, no. (%)	47 (56%)	41 (59%)	6 (43%)	0.28
Diabetes mellitus, no. (%)	11 (13%)	8 (11%)	3 (21%)	0.31
Coronary artery disease, no. (%)	10 (12%)	10 (14%)	0 (0%)	0.13
CHA_2_DS_2_VASc score 0 or 1, no. (%)	40 (47%)	34 (48%)	6 (43%)	0.55
Use of any anticoagulant drug, no. (%)	52 (62%)	43 (61%)	9 (64%)	0.84
Use of any antiarrhythmic drugs, no. (%)	54 (64%)	46 (66%)	8 (57%)	0.54
Use of flecainide or propafenone, no. (%)	29 (35%)	22 (31%)	7 (50%)	0.18
Use of sotalol >160 mg/day, no. (%)	20 (24%)	19 (27%)	1 (7%)	0.11
Use of amiodarone, no. (%)	5 (6%)	5 (7%)	0 (0%)	0.30
Left ventricular ejection fraction, mean ± SD	57 ± 2	57 ± 2	56 ± 3	0.10
Left atrial volume, mean ± SD	31 ± 7	31 ± 8	29 ± 7	0.29

### 3.2 PV diameters

**Figure 1:**
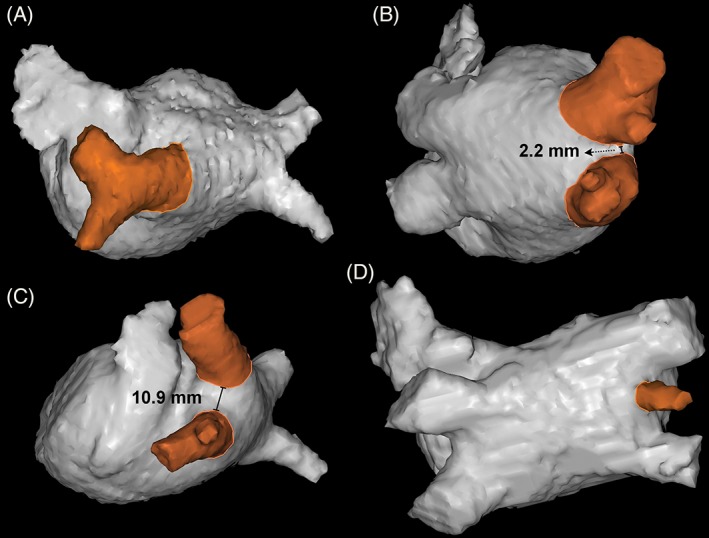
Examples of pulmonary vein anatomy. A, Left common pulmonary vein, B, shared carina, C, non shared carina, and D, right middle pulmonary vein

Measures of interobserver reproducibility revealed that all ICC estimates were significant (data not shown), except for three variables. In these instances, not enough (n = 3) measurements were made to produce reproducible results. Figure 1 shows examples of PV anatomy. In total, 17 (20.2%) of patients had a left common PV (Figure 1A). Fifty‐one patients (61%) had a shared carina left, 35 patients (42%) had a shared carina right (Figure 1B). Nine patients (11%) were classified having a right middle PV (RMPV) (Figure 1D). Table 2 displays all mean PV measurements including calculated ovality. Maximal diameters of LSPV, LIPV, RSPV, and RIPV were respectively 19.5 (17.1‐20.8), 17.4 (16.0‐18.9), 20.5 (18.3‐23.5), and 18.0 (15.8‐20.6) mm.

**Table 2:** Pulmonary vein diameters

	No recurrence (n = 70)	Recurrence (n = 14)	*P*‐value
Left sided PV			
LSPV max outer diameter, median (IQR)	20.8 (18.3‐22.5)	20.8 (18.5‐23.0)	0.83
LIPV max outer diameter, median (IQR)	18.5 (17.1‐20.0)	17.5 (16.7‐19.0)	0.19
Right sided PV			
RSPV max outer diameter, median (IQR)	23.4 (20.5‐25.9)	22.4 (20.5‐26.1)	0.74
RIPV max outer diameter, median (IQR)	20.2 (17.3‐22.2)	19.1 (18.6‐21.0)	0.49

Abbreviations: IQR, interquartile range; LIPV, left inferior pulmonary vein; LSPV, left superior pulmonary vein; PV, pulmonary vein; RIPV, right inferior pulmonary vein; RSPV, right superior pulmonary vein.

### 3.3 AF recurrence

At 12 months, 14 patients (17%) had experienced AF recurrence. When compared to patients without recurrence, there was no observed difference in ovality index in patients with AF recurrence (Table 3). Also, no significant interaction between a shared carina left or right, or left common PV and AF recurrence at 12 months (Table 2) was observed. No significant difference in AF recurrence was observed between presence and absence of RMPV (17% vs 11%, respectively, *P* = 0.64).

**Table 3:** Ovality index

		No recurrence (n = 70)	Recurrence (n = 14)	*P*‐value
LSPV ovality	Round	13 (19%)	2 (14%)	0.85
	Oval	22 (32%)	4 (29%)	
	Flat	34 (49%)	8 (57%)	
LIPV ovality	Round	13 (19%)	3 (21%)	0.39
	Oval	22 (32%)	2 (14%)	
	Flat	33 (49%)	9 (64%)	
RSPV ovality	Round	29 (42%)	6 (43%)	0.23
	Oval	22 (32%)	7 (50%)	
	Flat	18 (26%)	1 (7%)	
RIPV ovality	Round	42 (62%)	13 (93%)	0.076
	Oval	21 (31%)	1 (7%)	
	Flat	5 (7%)	0 (0%)	

Abbreviations: LIPV, left inferior pulmonary vein; LSPV, left superior pulmonary vein; RIPV, right inferior pulmonary vein; RSPV, right superior pulmonary vein;.

### 3.4 Cox proportional hazard analysis

Univariate Cox regression (data not shown) showed that BMI (HR = 0.91, 95% confidence interval [CI] = 0.79‐1.05), usage of sotalol >160 mg/day (HR= 0.24, 95% CI = 0.03‐1.80), LVEF (HR = 0.86, 95% CI = 0.70‐1.05) and LAVI (HR = 0.94, 95% CI = 0.86‐1.01) were considered for entrance in our multivariable model. After multivariate analysis none were significant. Table 4 shows the Cox regression for the vein diameters, ovality index, shared carina, or morphological characteristics of which none were considered a significant predictor of AF recurrence.

**Table 4:** Cox regression analysis of pulmonary vein anatomy

	Hazard ratio	95% CI	*P*‐value
Shared carina left	0.88	0.30‐2.53	0.810
Shared carina right	0.55	0.17‐1.77	0.318
LSPV outer ovality			
Round	1	1	—
Oval	1.84	0.37‐9.10	0.457
Flat	1.16	0.23‐5.74	0.856
LIPV outer ovality			
Round	1	1	
Oval	0.69	0.12‐4.12	0.682
Flat	1.25	0.27‐5.79	0.774
RSPV outer ovality			
Round	1	1	—
Oval	0.93	0.32‐2.69	0.89
Flat	—	—	—
RIPV outer ovality			
Round	1	1	—
Oval	—	—	—
Flat	—	—	—

Abbreviations: CI, confidence interval; LIPV, left inferior pulmonary vein; LSPV, left superior pulmonary vein; RIPV = right inferior pulmonary vein; RSPV, right superior pulmonary vein .

## 4 DISCUSSION

In the present study, we explored whether specific characteristics of PV dimension or anatomy could predict outcome of cryoballoon ablation in patients with paroxysmal AF. Neither PV ovality, the presence of anatomical variants (right middle PVs, common ostia), shared carina nor carina width influenced AF recurrence rate at 12 months.

As PVI outcome is difficult to predict because of many confounding factors, we limited our analysis to a homogenous patient population and reproducible ablation procedure, that is, paroxysmal AF patients treated with a 28‐mm second‐generation cryoballoon. Other studies evaluating the predictive value of PV dimensions and morphology on ablation outcome used both the first‐ and second‐generation balloons, different sizes balloon (23‐ and 28‐mm) and a heterogeneous group of patients (both paroxysmal and/or persistent AF). In concordance with previous studies, we found that 11% of patients had a right middle PV.^8^, ^14^ There was no association between the presence of a right middle PV and AF recurrence. This has also been reported in a comparable study (The Sustained Treatment of Paroxysmal Atrial Fibrillation (STOP AF) trial.^15^ In this trial, early or late recurrence was not associated with non‐standard anatomy following cryoballoon PVI.^15^ Another theory is that early branching of the RLPV predicts AF recurrence.^16^ Several other studies have assessed PV morphology and success of cryoballoon ablation.^9^, ^11^, ^13^ Principle findings were that left‐side PV ovality is more often observed than right‐sided ovality and that left‐sided ovality is associated with more difficult isolation of the vein and more AF recurrence. We observed no difference for any PV diameter or ovality index. The difference between our study and previous studies was a more heterogeneous patient population including persistent and paroxysmal AF patients or usage of a first‐generation cryoballoon. The presently used second‐generation balloon has double the amount of cooling ports and the balloon has a more evenly spread cooling zone. We hypothesized that ablation with the first‐generation cryoballoon in oval‐shaped PVs not all the tissue is affected by the cryoapplication. This potentially may lead to higher recurrence rates. The more powerful second‐generation cryoballoon used in this study could explain why PV ovality does not influence cryoballoon ablation success rate anymore. This was also demonstrated in a recent study in patients with a left common PV.^17^ In the present study, using a second‐generation cryoballoon, these patients had (compared with a control population) similar high acute success rates and comparable durable results.^17^

AF triggers often originate from the right and left PV carina.^18^ Through histological and electrophysiological studies, the importance of the carina in PVI was demonstrated.^19^, ^20^, ^21^ Crossing myocardial strands and bridges at the interpulmonary isthmus may be the anatomical substrate for electrical connection between superior and inferior PVs.^20^ However, no studies investigated the role of the carina width for predicting cryoballoon ablation success. Cryoapplications at ipsilateral PVs result in multiple (at least 4 if 2 application are given per vein) applications at the carina. In patients with small width carina there is overlap of the cryoapplications. This may have impact on durability of cryolesions and also on recurrence rate. We, however, found no difference in carina width in patients with or without AF recurrence.

### 4.1 Limitations

Our study is limited by the limitations inherent to the retrospective study design. Recurrences of AF were monitored by standard out‐patient clinic ECG and Holter monitoring, asymptomatic episodes of AF will therefore been missed. Also, all PV diameters have been measured using a manual caliper function. A (semi‐)automated caliper function would produce more reliable and consistent results. The most reliable results would have been produced through an algorithm that calculates the exact location where the balloon touches the ostium, based on the pre‐procedural CT. Furthermore, we did not account for PVs with early branching.

## 5 CONCLUSION

No specific characteristics of PV dimensions or morphology were associated with AF recurrence after cryoballoon PVI in patients with paroxysmal AF. Further research on individual parameters associated with the success or failure of PVI is essential.

## CONFLICTS OF INTEREST

The authors declare no potential conflict of interests.
